# Influenza and COVID-19 Vaccination Rates Among Children Receiving Long-Term Ventilation

**DOI:** 10.1001/jamanetworkopen.2024.30989

**Published:** 2024-08-28

**Authors:** Robert J. Graham, Luis F. Enriquez, Angelo F. Elmi, James V. Zavadoski, Lisa G. Bielak, Christopher D. Baker, Jonathan M. Mansbach

**Affiliations:** 1 Department of Anesthesiology, Critical Care and Pain Medicine, Division of Critical Care Medicine, Boston Children’s Hospital, Boston, Massachusetts; 2 Department of Pediatrics, Boston Children’s Hospital, Boston, Massachusetts; 3 Department of Biostatistics and Bioinformatics, Milken Institute School of Public Health at the George Washington University, Washington, DC; 4 Clinical Research Operations Center, Boston Children’s Hospital, Boston, Massachusetts; 5 Department of Pediatrics, Section of Pulmonary and Sleep Medicine, University of Colorado School of Medicine, Aurora

## Abstract

This cohort study examines rates of vaccination for COVID-19 and for influenza among children receiving long-term home ventilation.

## Introduction

Children who receive tracheostomy and home ventilation (HV) are at high risk of acute respiratory infections (ARIs), the leading cause of hospitalization and death among these patients.^1^ The Centers for Disease Control and Prevention and the American Academy of Pediatrics recommend annual influenza and COVID-19 vaccinations for all children aged 6 months or older, unless medically contraindicated.^2,3^ Concern, however, exists that growing antivaccination sentiments, emerging “medical freedom movements,” and specific mistrust of COVID-19 vaccines^4^ may negatively affect rates of vaccination among children at high risk. Given these concerns, we examined rates of influenza and COVID-19 vaccination among children receiving long-term HV. We hypothesized that uptake of the influenza vaccine would be higher than COVID-19.^5^

## Methods

From March 2022, to October 2023, the 13-center (12 states and the District of Columbia) Multicenter Tracheostomy Collaboration (MyTRaCh) study enrolled 193 children receiving transtracheal HV into a prospective cohort study and collected longitudinal tracheal aspirates to examine the pathobiology of ARIs. The Boston Children’s Hospital institutional review board approved this study. Twelve collaborating sites had reliance and data-use agreements. We excluded children with cystic fibrosis, immunodeficiencies, and primary ciliary dyskinesia; those living in long-term care facilities; and, for this analysis, those ineligible for vaccination based on age at enrollment. Site teams collected demographics, race and ethnicity data, medical history, and immunization data by structured caregiver interview and reconciliation with available records. Race and ethnicity were extracted from chart review and self-identified during parent-reported screening. Race and ethnicity were collected to better describe the cohort and as part of the analysis plan in compliance with National Institutes of Health/National Institute of Allergy and Infectious Diseases requirements for the larger microbiome study. We conducted descriptive statistics and multivariable logistic regression using generalized estimating equations to account for clustering by center to analyze the association between demographics and vaccination status (eMethods in Supplement 1). Findings were reported in accordance with the STROBE reporting guideline. The significance threshold was *P* < .05 using a 2-sided test. All data management and analysis were conducted using SAS, version 9.4.

## Results

Of 313 participants, we confirmed complete data for 282 (90%), and 193 (62%) were age eligible for vaccination. The median age was 10.51 years (IQR, 6.26-14.1 years) with a median age of tracheostomy of 6.0 months (IQR, 2.8-48 months). Sixty-three percent were male, and 58% were non-Hispanic White. A total of 145 (75%) received the influenza vaccine either annually or most years. A total of 102 (53%) completed their initial COVID-19 vaccine series; however, only 45 participants (23%) received the recommended booster. Among children who received an influenza vaccine, 96 (61%) also received a COVID-19 vaccine; the decreasing frequency of COVID-19 vaccination corresponded with that of the influenza vaccine. Among 35 participants (18%) who never received an influenza vaccine, 6 (17%) received a COVID-19 vaccine (Table 1). Our multivariable analysis adjusting for demographics and clustering by site found that those with significantly higher odds of receiving any COVID-19 vaccine were older (odds ratio [OR], 1.14; 95% CI, 1.09-1.19) and had a previous influenza vaccine (OR, 8.93; 95% CI, 4.59-17.37) (Table 2).

**Table 1.** Characteristics of 193 Children With Tracheostomy and Home Ventilation in the Multicenter Tracheostomy Collaboration Study by COVID-19 Vaccination Status^a^

Characteristic	COVID-19 initial vaccine series (n = 102)	No COVID-19 vaccine (n = 91)	*P* value
Age, median (IQR), y	12.27 (7.99-15.84)	7.58 (4.23-12.24)	<.001
Age at placement of tracheal tube, median (IQR), y	0.44 (0.24-4.22)	0.56 (0.21-3.70)	.87
Sex assigned at birth, No. (%)			
Male	67 (55)	54 (45)	.36
Female	35 (49)	37 (51)	
Race and ethnicity, No. (%)			
Hispanic	21 (47)	24 (53)	.30
Non-Hispanic Black	13 (65)	7 (35)	
Non-Hispanic White	62 (55)	50 (45)	
Other^b^	6 (38)	10 (62)	
Health insurance, No. (%)			
Private	8 (47)	9 (53)	.03
Public	42 (43)	55 (57)	
Private and public, No. (%)	39 (65)	21 (35)	
Unknown	13 (68)	6 (32)	
Flu vaccine, No. (%)			
Yes	96 (61)	62 (39)	<.001
No	6 (17)	29 (83)	
Flu vaccine regularity, No. (%)			
Every year	77 (66)	39 (34)	.03
Most years	15 (50)	15 (50)	
Once or twice	3 (30)	7 (70)	
Unknown	1 (50)	1 (50)	
None (n = 35 [18%])^c^			
COVID-19 booster, No. (%)			
Yes	45 (100)	0	<.001
No	55 (38)	91 (62)	
Missing (n = 2 [1%])	2	0	

^a^ Percentages are reported as row percentages.

^b^ Other included American Indian, Alaska Native, Asian, Native Hawaiian, Other Pacific Islander, or other not specified.

^c^ Thirty-five participants did not receive any influenza vaccine; 6 of those received the initial COVID vaccine. This cohort was not included in analysis of influenza vaccine frequency and COVID vaccination.

**Table 2.** Univariable and Multivariable Association Between Participant Characteristics and Initial COVID-19 Vaccination Series Status Among 193 Children in the Multicenter Tracheostomy Collaboration Study

Characteristic	Model			
	Univariable model		Multivariable model	
	OR (95% CI)	*P* value	OR (95% CI)	*P* value
Age^a^	1.13 (1.08-1.17)	<.001	1.14 (1.09-1.19)	<.001
Sex assigned at birth				
Female	0.77 (0.39-1.51)	.44	0.53 (0.24-1.17)	.12
Male	1.00 [Reference]		1.00 [Reference]	
Race and ethnicity				
Non-Hispanic White	0.93 (0.54-1.61)	.80	0.89 (0.45-1.73)	.73
Hispanic, non-Hispanic Black, and other^b^	1.00 [Reference]		1.00 [Reference]	
Health insurance^c^				
Private only	0.58 (0.2-1.67)	.08	0.62 (0.25-1.56)	.10
Public only	0.48 (0.2-1.16)		0.51 (0.22-1.14)	
Unknown	1.04 (0.13-8.12)		2.13 (0.38-12.09)	
Both public and private	1.00 [Reference]		1.00 [Reference]	
Influenza vaccination status				
Yes	6.97 (2.85-17.09)	<.001	8.93 (4.59-17.37)	<.001
No	1.00 [Reference]		1.00 [Reference]	

^a^ Age is a continuous variable.

^b^ Other includes American Indian, Alaskan Native, Asian, Native Hawaiian, Other Pacific Islander, or other not specified.

^c^ Health insurance 95% CIs were calculated with a Bonferroni correction for the 4 subcategories.

## Discussion

Children with tracheostomy and HV are at high risk of morbidity and mortality from viral ARIs. Among MyTRaCh enrollees, we found significant discrepancy between influenza and COVID-19 vaccination status. Compared with immunization status overall among US children aged younger than 18 years, our cohort had higher adherence to influenza vaccination (75% vs 50%), similar rates of initial COVID-19 vaccination (53% vs 50%), and much lower rates of receiving a COVID-19 booster (23% vs 50%).^5,6^

MyTRaCh families have successfully integrated complex technology support (eg, tracheostomy tubes, home ventilators, gastrostomy tubes, time-consuming medical care regimens) into their lives and understand their children’s vulnerabilities. Their engagement with tertiary care centers (ie, HV programs), primary care, and home care nursing suggests that opportunities for screening, education, and access were not barriers to vaccination. Unfortunately, the low vaccination rates in this study population suggest that COVID-19 vaccine hesitancy is apparent for adults making decisions for this very high-risk group of children.^5^

This study’s limitations included (1) reliance on parental reporting as the primary data source, although caregivers may overestimate vaccination status; (2) uncertainty of concordance of influenza and COVID-19 vaccination in a given season; and (3) failure to ask questions about absolute contraindications to vaccination or reason for not vaccinating.

Ultimately, there is discordance between the intensive, technology-dependent care these families provide their children, their relative acceptance of influenza vaccine, and the low COVID-19 vaccination rates. These findings highlight the need for qualitative studies to understand COVID-19 vaccine resistance, systematic measures to optimize access, and additional education programs among populations at high risk and the general public.

## Notes

Supplement 1. **eMethods.** Multivariate Modeling Methods

Supplement 2.
Data Sharing Statement
