# Supplementary material for: Influenza and COVID-19 Vaccination Rates Among Children Receiving Long-Term Ventilation
Source: JAMA Netw Open. 2024 Aug 28;7(8):e2430989. doi: 10.1001/jamanetworkopen.2024.30989 (PMC11358855; doi:10.1001/jamanetworkopen.2024.30989)
Supplement: Supplement 1. — eMethods. Multivariate Modeling Methods [file jamanetwopen-e2430989-s001.pdf]

Supplemental Online Content

Graham RJ, Enriquez LF, Almi AF, et al. Influenza and COVID-19 vaccination rates among children receiving long-term ventilation. *JAMA Netw Open*. Published online: 2024;7(8):e2430989. doi: 10.1001/jamanetworkopen.2024.30989

eMethods: Multivariate Modeling Methods

This supplemental material has been provided by the authors to give readers additional information about their work.

© 2024 Graham RJ et al. *JAMA Netw Open*.

Multivariate Modeling Methods:

All models were fit using Generalized Estimating Equations (GEE) logistic regression controlling for clustering by clinical center with an exchangeable correlation structure. This assumes that there is a correlation in the outcome of all individuals who are in the same clinical center. The standard errors from the GEE model are adjusted using a robust sandwich covariance estimate that incorporates the exchangeable correlation structure into the estimates and provides inferences that do not assume subjects in the study are mutually independent.

The univariable models estimate the effects of each variable in the rows of the table without controlling for the other variables in the table. They do, however, control for the correlation within centers. The multivariable models control for all other variables in the rows of the table as well as for clustering by center. There are no other variables that we controlled for beyond what is shown in Table 2.

© 2024 Graham RJ et al. *JAMA Netw Open*.
