# Supplementary material for: Influenza and COVID-19 Vaccination Rates Among Children Receiving Long-Term Ventilation
Source: JAMA Netw Open. 2024 Aug 28;7(8):e2430989. doi: 10.1001/jamanetworkopen.2024.30989 (PMC11358855; doi:10.1001/jamanetworkopen.2024.30989)
Supplement: Supplement 2. — Data Sharing Statement [file jamanetwopen-e2430989-s002.pdf]

Data Sharing Statement

Graham. Influenza and COVID-19 Vaccination Rates Among Children Receiving Long-Term Ventilation. *JAMA Netw Open*. Published August 28, 2024.
doi:10.1001/jamanetworkopen.2024.30989

Data
Data available: Yes
Data types: Deidentified participant data
How to access data: Direct contact with the first and senior authors, RJG and JMM.
When available: With publication

Supporting Documents
Document types: None

Additional Information
Who can access the data: Those with a DUA and CCI/IRB approved protocol.
Types of analyses: As requested
Mechanisms of data availability: Signed DUA will be needed to comply with the IRB.
